# Acoustofluidic Measurements on Polymer-Coated Microbubbles: Primary and Secondary Bjerknes Forces

**DOI:** 10.3390/mi9080404

**Published:** 2018-08-15

**Authors:** Gianluca Memoli, Kate O. Baxter, Helen G. Jones, Ken P. Mingard, Bajram Zeqiri

**Affiliations:** 1 School of Engineering and Informatics, University of Sussex, BN1 9QJ Falmer, UK; 2 National Physical Laboratory, TW11 0LW Teddington, UK; kate.baxter@npl.co.uk (K.O.B.); Helen.jones@npl.co.uk (H.G.J.); Ken.mingard@npl.co.uk (K.P.M.); bajram.zeqiri@npl.co.uk (B.Z.)

**Keywords:** microbubbles, acoustofluidics, Bjerknes forces, compressibility, 43.25.Yw, 47.35.Rs, 43.25.Qp, 43.25.+y, 62.20.mq

## Abstract

The acoustically-driven dynamics of isolated particle-like objects in microfluidic environments is a well-characterised phenomenon, which has been the subject of many studies. Conversely, very few acoustofluidic researchers looked at coated microbubbles, despite their widespread use in diagnostic imaging and the need for a precise characterisation of their acoustically-driven behaviour, underpinning therapeutic applications. The main reason is that microbubbles behave differently, due to their larger compressibility, exhibiting much stronger interactions with the unperturbed acoustic field (primary Bjerknes forces) or with other bubbles (secondary Bjerknes forces). In this paper, we study the translational dynamics of commercially-available polymer-coated microbubbles in a standing-wave acoustofluidic device. At increasing acoustic driving pressures, we measure acoustic forces on isolated bubbles, quantify bubble-bubble interaction forces during doublet formation and study the occurrence of sub-wavelength structures during aggregation. We present a dynamic characterisation of microbubble compressibility with acoustic pressure, highlighting a threshold pressure below which bubbles can be treated as uncoated. Thanks to benchmarking measurements under a scanning electron microscope, we interpret this threshold as the onset of buckling, providing a quantitative measurement of this parameter at the single-bubble level. For acoustofluidic applications, our results highlight the limitations of treating microbubbles as a special case of solid particles. Our findings will impact applications where knowing the buckling pressure of coated microbubbles has a key role, like diagnostics and drug delivery.

## 1. Introduction

The use of acoustic forces is an established technique for manipulating and sorting cells [1,2,3], micro-particles and micro-droplets in lab-on-a-chip applications [4]. Applications exploit the interaction of the suspended solid particles with the acoustic field, such as acoustic radiation force and streaming, to exert control on their dynamics [4] and measure their elastic properties [3]. The acoustic manipulation of microbubbles (MBs) is a more recent application [5,6], which builds on the success of coated microbubbles as a contrast agent in ultrasound imaging [7] and focuses on their use in drug delivery [8,9] and bio-sensing applications [10]. Microstreaming generated from the oscillations of uncoated microbubbles (acoustically, thermally or chemically actuated) has been used for localised flow control, leading to dynamic switching in microfluidic chips [11,12], microswimmers’ propulsion [13,14] and localised probing of cell properties [15,16]. In these applications, microbubbles are potentially more effective than particles because their deformation can be controlled by an external agent, but they are often simply treated as particles with a higher compressibility.

According to theory [17,18], however, the behaviour of microbubbles is frequency-dependent: uncoated microbubbles in a watery solution would behave according to classical Gor’kov theory [19] only when excited below their acoustic resonance. In these conditions, the forces are similar to the ones acting on solid particles, but opposite in sign [20,21,22]. In the presence of a standing wave, MBs would therefore move towards antinodes when solid particles of similar size would move towards nodes [19]. Above resonance, the sign of the force changes, and bubbles move to nodes, just like solid particles of a similar size [5,6]. In addition, the response of microbubbles in an acoustofluidic device depends on their number concentration: ultrasound-activated interactions between bubbles and surrounding objects (e.g., other bubbles) depend on inter-bubble distance and are predicted to be stronger than for particles of equivalent diameter [17], leading to concentration-dependent scattering [23]. Finally, the presence of a coating on the shell of bubbles leads to pressure-dependent phenomena, i.e., onset of oscillations, buckling and rupture [24,25,26]. All these phenomena need to be quantified for the optimisation of applications based on acoustically-driven microbubble dynamics.

This study presents a set of experiments using commercially-available polymer-coated MBs, highlighting the dependence of microbubble’s compressibility on excitation frequency, pressure and number concentration. Experiments are conducted in a well-known acoustofluidic manifold (described in [27]), at increasing acoustic driving pressures and two different number concentrations. First, we look at isolated bubbles in their motion towards an “aggregation site” (i.e., the closest acoustic antinode, in our experimental conditions), under the action of radiation forces (primary Bjerknes forces). In this way, we obtain a value of the effective acoustic pressure $p_{\mathrm{meas}}$ acting on the bubbles, as a function of the driving voltage at two microbubble concentrations. Second, we track pairs of bubbles joining into a single entity (i.e., a “doublet”) before proceeding further towards the aggregation site and following the example of Garcia-Sabatè et al. [18], we use these events to measure bubble-bubble interactions (i.e., secondary Bjerknes forces) perpendicular to the direction of motion. Third, we observe doublets aggregating into linear structures, perpendicular to the direction of motion of the bubbles (“ripples”), and discuss their separation using recent [17] and historical [28,29] theories, to estimate the nature of the interaction between bubbles in the direction of motion. In doing so, we show that the forces acting on the bubbles depend on the acoustic pressure, but not as expected by particle-based theories [17], which assume a purely elastic behaviour. In particular, we show evidence of a threshold pressure, above which phenomena classically attributed to bubble-bubble interactions (secondary Bjerknes forces [17]) can be observed. We propose a correction to the acoustic contrast factor to account for secondary Bjerknes forces and summarise our findings in a dynamical measurement of the compressibility of coated microbubbles: a key parameter for the uptake of microbubble-based therapies [8] and sensing applications [15,16]. Thanks to a direct estimation of key shell parameters—obtained by milling and compressing a selection of bubbles under a Focussed Ion Beam Scanning Electron Microscope (FIB-SEM)—we discuss our results in terms of the onset of volume oscillations [25,30] and of buckling [26].

## 2. Materials and Methods

**Figure 1:**
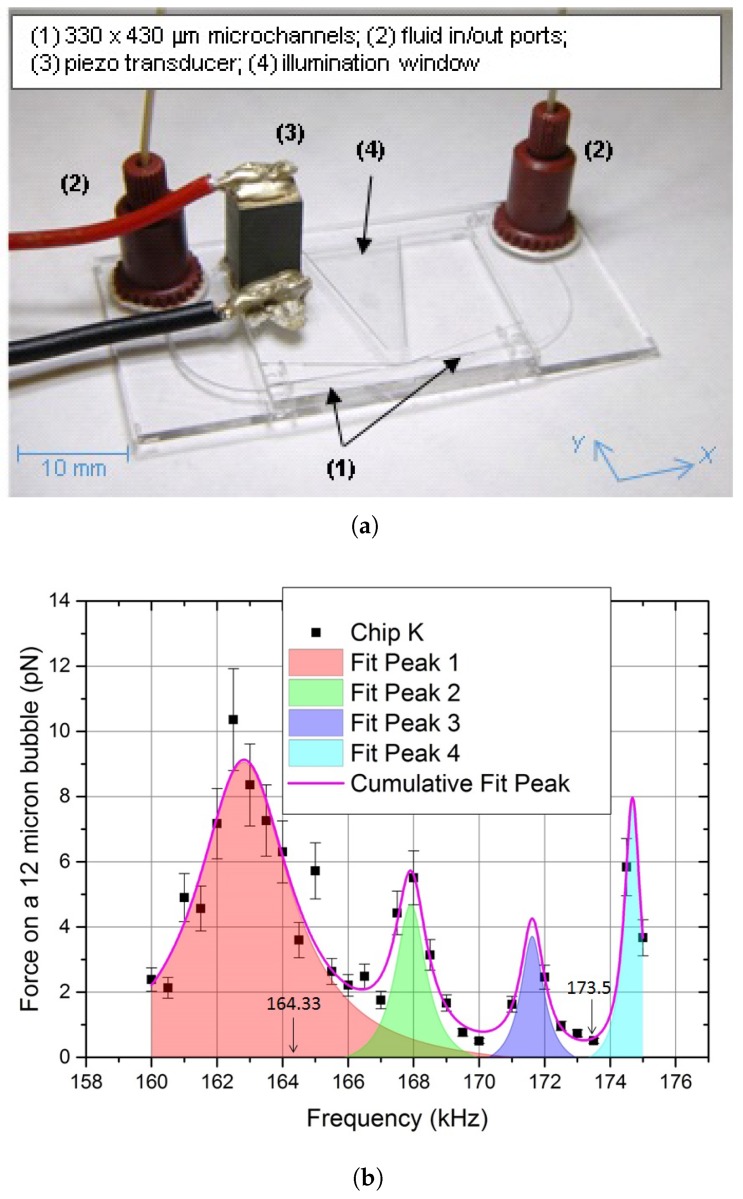
Experimental setup: (**a**) the microfluidic chip used in this work and (**b**) its response to frequency, in terms of the force measured on a 12-μm diameter Expancel${}^{\mathrm{TM}}$ bubble, as reported in [27]. Each point reports the average over 5 isolated bubbles (see below). Also highlighted in (a) are the directions of the reference axes, with the $\hat{X}$ along the main channel and the $\hat{Y}$ perpendicular to it. The origin of the coordinates was set at the start of the channel, on the side where the piezoelectric transducer is positioned.

Experiments were conducted in a glass microfluidic chip (W: 25 mm, H: 2 mm, L: 20 mm), designed at the National Physical Laboratory (NPL) and manufactured by Dolomite Microfluidics (Royston, UK). The microfluidic chip (see [27] for full details) presents a K-shaped manifold of etched microchannels (330 μm × 430 μm section, with a 100 μm-wide flat surface on the top and bottom of each channel) and is mounted on a glass base (W: 40 mm, H: 1 mm, L: 25 mm), which provides fluidic connection to the in/out ports. A trapezoidal window provides for lateral illumination of the central area (see Figure 1a). The thickness of the polished flat surface above the trapping region was 0.17 mm [31]. The K-shaped geometry has been chosen to facilitate future studies, where the two inclined channels will be used for monitoring acoustic emission from trapped bubbles.

The acoustic field was generated using a 5.9 mm × 5.9 mm × 13 mm Lead Zirconate Titanate (PZT) transducer (Morgan Ceramics Ltd., Southampton, UK) with a nominal resonance (in air) of ∼164 kHz, bonded onto the device’s top surface using conductive epoxy (CircuitWorks${}^{\mathrm{TM}}$ CW2400, Farnell, Leeds, United Kingdom). A sinusoidal voltage in the range of 160–180 kHz was used to drive the PZT transducer, after being amplified using a chain formed by a signal generator (Agilent 33250A, Agilent Technologies, Santa Clara, CA, USA), a power amplifier (E&I, Model A300, Electronics & Innovation, Rochester, NY, USA) and a 1:25 step-up matching impedance transformer.

As shown in Figure 1b, which reports the force spectrum for a typical realisation of the microchip, we found the force on a 12 μm diameter Expancel${}^{\mathrm{TM}}$ bubble to depend on frequency, due to the presence of different acoustic resonances [27]. The frequency of 164.33 kHz (highlighted in Figure 1b) was however the one where aggregation in the central area was repeatedly observed in different realisations of the chip: we consequently used this frequency for most of this study.

The acoustic pressure in this device has been accurately characterised (both spatially and as a function of the driving voltage) in previous studies [27] providing, at 164.33 kHz, a calibration coefficient of 47.8±0.8 Pa$\cdot {{\mathrm{mV}}_{\mathrm{pp}}}^{-1}$ (peak pressure, 1 standard deviation) for input voltages up to 85 mV${}_{\mathrm{pp}}$ (i.e., input peak pressures up to 4.1 kPa). Direct force measurements in the central area of the main microfluidic channel confirm the sinusoidal nature of the acoustic field at this frequency, with an antinode in the centre [27].

The maximum power registered by the power amplifier during these experiments was 7 W, far below the amplifier’s rated specifications: therefore the amplifier chain was assumed to be working linearly. A maximum temperature increase of 1.5 ${}^{\circ }$C was observed on the surface of the chip (using a thin-film thermocouple) during tests (i.e., with the field ON), but the amount of vibrational energy transmitted to the main channel does not suggest localised heating. After each experiment, the temperature was allowed to return to its initial value. For this study, then, we assumed the fluid temperature to be constant and equal to the ambient value of $20\pm 1\;{}^{\circ }$C (experiments were run in a temperature-controlled room).

Similarly, since a maximum change of 0.3% in the speed of sound has been observed in the presence of Expancel${}^{\mathrm{TM}}$ concentrations of $∼10^{6}$ bubbles/mL at MHz frequencies [23], we consider the speed of sound unaffected by the bubbles in this work. Under these assumptions, we consider the driving acoustic field in the microfluidic device, $p_{a}$, unperturbed by the presence of the bubbles.

### 2.1. Microbubble Characterisation

In this study, we use different number concentrations of commercially-available polymeric-shelled bubbles (Expancel${}^{\mathrm{TM}}$ WU-20; gas: iso-butane; coating: acrylic copolymer, CAS 38742-70-0; diameter: 6–20 μm; manufactured by Akzo Nobel, Amsterdam, NL, USA). Polymer-coated Expancel${}^{\mathrm{TM}}$ microbubbles were expanded by leaving them for 10 min in boiled water, mixed with a 0.08 M solution of sodium dodecyl sulfate (SDS) and injected into a microfluidic chip (see Section 2) using a 1-mL syringe, before closing the open sides of the channels with Vaseline${}^{\mathrm{TM}}$. In the experiments, we injected a diluted sample of Expancel${}^{\mathrm{TM}}$ microbubbles into the chip and switched on the acoustic standing wave, causing the bubbles to move towards an aggregation point (see the Supplementary Material for a movie).

Once in the chip, we recorded Expancel${}^{\mathrm{TM}}$ dynamics through a CCD camera (Model DCU223M, Thorlabs Ltd., Ely, UK) and an InfiniProbe TS-160 objective (Infinity, Franklin, TN, USA) in a brightfield microscopy setup. We calibrated the images using a 400-μm NPL graticule (National Physical Laboratory, Teddington, UK) and used a basic thresholding method in ImageJ (Fiji distribution [32]) to establish the diameter of each monitored bubble, thus allowing a measurement of their size distribution after expansion directly in the chip (measured mode diameter: 10.1 μm, measured average diameter: 12.4 μm, 10% percentile: 8.1 μm, 90% percentile: 18.1 μm). An uncertainty of ∼0.2 μm was assigned to this method, due to pixel resolution [27]. We chose to leave the microbubble sample as close to the commercial one as possible, and therefore, no further preparation of the bubbles (e.g., sieving, to reduce the width of their size distribution) was conducted. We used camera images also to assess number concentrations, in the range of $3\times 10^{5}$–$10\times 10^{5}$ bubbles/mL, by counting the bubbles appearing in the videos, as described by Grishenkov et al. [33]. In the experiments, we selected two different bubble concentrations: one where we expect interactions to be detectable ($10^{6}$ bubbles/mL, according to [23]) and one approximatively three-times lower, i.e., $3.5\times 10^{5}$ bubbles/mL.

At the start of this work, we estimated the resonance frequency $\omega_{s}=2\pi f_{s}$ of a given polymer-coated microbubble using the linearised Hoff model [22]:

(1) $$f_{s}=\frac{1}{2\pi R_{0}}\sqrt{\frac{1}{\rho_{l}}\left[3\gamma P_{0}+\frac{2(3\gamma -1)\sigma }{R_{0}}+\frac{4\chi }{R_{0}}\right]}$$

where $R_{0}$ is the equilibrium radius of the bubble, $\rho_{l}$ is the liquid density, γ is the ratio of the specific heats of the gas inside the bubble, $P_{0}$ is the hydrostatic pressure, σ is the surface tension at the shell-liquid interface and χ is the elasticity parameter of the shell. In the case of a polymer-coated bubble, Hoff et al. [22] proposed to use for χ the expression for incompressible flat sheets: $\chi_{2D}=3E_{s}d_{s}/\left(2\left(1+2\nu \right)\right)$ with $E_{s}$ and ν respectively the Young’s modulus and Poisson’s ratio of the material in the shell and $d_{s}$ its thickness. Using for the shell typical properties from the literature ($P_{0}=$ 101 kPa, γ= 1.07, Young’s modulus: $E_{s}=3\pm 0.3$ GPa, Poisson’s ratio ν= 0.3, shell thickness $d_{s}\geq$ 3 nm [34,35]) and $\sigma =33\;\mathrm{mN}\cdot m^{-1}$ for a 0.08 M water-SDS solution [36], the resonant frequency for the range of Expancel${}^{\mathrm{TM}}$ diameters utilised in this study was calculated to be above 1 MHz; see Equation (1). It was therefore not surprising to see Expancel${}^{\mathrm{TM}}$ bubbles moving towards antinodes at the frequencies used in this study, in the range 160–180 kHz (see also Appendix C).

### 2.2. Primary Bjerknes Forces

In the experiments with isolated bubbles, run at 164.33 kHz and aimed at measuring the forces acting on isolated bubbles as a function of driving pressure (i.e., primary Bjerknes forces), we injected a diluted sample of Expancel${}^{\mathrm{TM}}$ microbubbles into the chip and switched on the acoustic standing wave, causing the bubbles to move towards an aggregation point (see Supplementary Movie).

We selected at least 5 different bubbles for each experimental condition (defined by frequency and unperturbed acoustic pressure $p_{a}$) and recorded their trajectories using the MTrackJ plugin in ImageJ [37]. Selected bubbles were isolated (i.e., at least 5 particle diameters from another bubble) and far (i.e., at least 20 bubble diameters) from the centre of the aggregation area. As the voltage was increased, it was necessary to take more repeats due to the presence of acoustic streaming, in the form of vortices detaching from the junction between the two “legs” of the K-shaped manifold. For each movie, the coordinate system was set at the centre of the aggregation point. Trajectories were fitted using a least-squares method, imposing a balance between the radiation force $F_{\mathrm{rad}}$ and the drag $F_{\mathrm{drag}}$. Having assumed a constant value for the acoustic contrast factor (Φ=−6753, see below), this left us with a single fitting parameter $p_{\mathrm{meas}}$ that we defined as the “effective” pressure: a sum of the input pressure $p_{a}$ and of the pressure scattered by the other bubbles [19,27,38]. Good agreement to the fits (i.e., $R^{2}∼0.9$) was obtained in all cases. Unless explicitly stated otherwise, the value of $p_{\mathrm{meas}}\left(f,p_{a},\Xi \right)$ assigned to each experimental condition (i.e., *f*: driving frequency; $p_{a}$: input acoustic pressure; Ξ: number concentration) was eventually a weighted average over the analysed trajectories (each corresponding to a different bubble diameter). This method is well established in the literature when particles are involved [4] and has been extended, by Memoli et al. [27], to Expancel${}^{\mathrm{TM}}$ microbubbles of diameter 10±2 μm at low number concentrations and input pressures below 1.5 kPa. [27].

In this work, we used for $F_{\mathrm{rad}}$ the classical expression from the Gor’kov model [17,19], but modified to take into account the resonant behaviour of the bubbles [20,21].

In the simple case of a spherical particle in a sinusoidal standing wave $p\left(x\right)=p_{0}\cos\left(kx\right)$, in the Rayleigh regime, this approach gives (primary Bjerknes force):

(2) $$F_{\mathrm{rad}}=\frac{4}{3}\pi \Phi \left(\tilde{\kappa },\tilde{\rho }\right)ka^{3}\frac{p_{0}^{2}}{2\rho_{l}c_{l}^{2}}\sin2kx\cdot \frac{\omega_{s}^{2}-\omega^{2}}{\left({\left(\omega_{s}-\omega \right)}^{2}+{\left(2\beta_{\mathrm{tot}}\omega \right)}^{2}\right)}$$

where $p_{0}\geq p_{a}$ is the “effective” acoustic pressure (sum of input acoustic pressure and scattered pressure), $p_{a}$ is the input acoustic pressure (not affected by the presence of the MBs at the concentrations used in this study), *a* is the particle radius, *k* is the wave number, $\tilde{\kappa }=\kappa_{p}/\kappa_{l}$ is the ratio between the compressibilities of the particle ($\kappa_{p}$) and the liquid ($\kappa_{l}$), $\tilde{\rho }=\rho_{p}/\rho_{l}$ is the ratio of their densities, $c_{l}$ is the speed of sound in the liquid medium, $\beta_{\mathrm{tot}}$ is the dampening coefficient for volume oscillations, ω=2πf is the frequency of the driving acoustic field and $\Phi =\frac{5\tilde{\rho }-2}{2\tilde{\rho }+1}-\tilde{\kappa }$ is also known as the acoustophoretic contrast factor (here calculated for a spherical particle). The change of sign implicit in Equation (2) was confirmed by the results of Rabaud et al. [39] and Bernassau et al. [6]: these authors also looked at the acoustic forces acting on confined bubbles, but used MBs with dimensions larger than the resonance radius ($\omega >\omega_{s}$), which therefore moved towards nodes of the field.

We used for $F_{\mathrm{drag}}$ the classical expression due to Stokes: $F_{\mathrm{drag}}=6\pi a\mu_{l}U_{b}$, where *a* is the bubble radius, $\mu_{l}$ is the viscosity of the liquid and $U_{b}$ is the bubble velocity [40,41]. For bubbles moving in surfactant-water solutions, this formula is valid in non-pure water when the Reynolds number is smaller than 0.1 [42]: a condition that was always met in our experiments. Rabaud et al. [39] showed, however, that it is necessary to correct the drag force to account for the presence of the walls of the microfluidic system: for bubbles always in contact with the walls, these authors proposed an additive correction proportional to $\xi^{1/5}$ for ξ>0.6 (ξ=(a/L), where *L* is the size of the microchannel). A more complex expression for the increased drag can be found in the classical text by Clift et al. [40], who for low Reynolds numbers and ξ<0.5 proposed as the leading term $\xi^{5}$. Under similar conditions, [38] proposed a leading correction $∼\xi^{2}$. The particular case of the interaction of bubbles with the walls of an acoustically-resonant pipe has been explored by Leighton [43], who showed an effect on the inertia and a different damping for bubbles moving, resulting in a change in their resonant frequency. In the experiments presented here, the additive correction to $F_{\mathrm{drag}}$ due to the walls (for the range of diameters considered) was estimated to be ≤0.5% for all theories and was therefore neglected.

Finally, in order to use $p_{\mathrm{meas}}$ as a single fitting parameter, we assumed the acoustic contrast factor in Equation (2) to be a known constant. In particular, we used Φ=−6753 for an uncoated spherical bubble of iso-butane in water (at $20\;{}^{\circ }$C, speed of sound in iso-butane: $204.3\;m\cdot s^{-1}$, density: 2.65 $\mathrm{kg}\cdot m^{-3}$, viscosity: $6.936\times 10^{-6}\;\mathrm{Pa}\cdot s$ [44], to be compared with Φ=−6222 of air and Φ=+0.15 of polystyrene).

Before fitting, we checked that the critical diameter above which the dynamics is radiation-dominated [38] was lower than the diameters used in this study (it was equal to 0.02 μm). Inertial effects due to streaming-induced velocities were therefore neglected.

### 2.3. Secondary Bjerknes Forces

For the experiments with multiple bubbles, aimed at determining the relative weight of bubble-bubble interactions (i.e., secondary Bjerknes forces) on bubble dynamics, we kept analysing a trajectory even when the relative bubble was no longer “isolated”, i.e., after the presence of other bubbles altered the path. We conducted two types of experiments:

1. At the single frequency of 164.33 kHz, but varying the input acoustic pressure $p_{a}$, we monitored the occurrence of “pairing”, defined as an event in which two previously isolated bubbles of similar size started interacting before joining into a single item (i.e., a “doublet”) and continuing to travel together towards the aggregation point. These events have been used by [18] to measure interactions between particles, perpendicular to the direction of motion.
2. In the range of 160–180 kHz, but at the fixed input voltage of 40 mV${}_{\mathrm{pp}}$, we observed the formation of lines of bubbles (“ripples”) perpendicular to the direction of motion. The formation of similar structures for particles has been used since the 19th Century to estimate the interaction forces acting in the direction of motion [28,29,45].

Self-aggregation into sub-wavelength structures has been observed for microbubbles [5,6,9,39] but, so far, always at frequencies above their resonance (i.e., when they move towards nodes). In particular, Rabaud et al. [5] observed the formation of structures they called “acoustic crystals” (using 20–50 μm-diameter bubbles within 25-μm channels at 70 and 220 kHz), while Bernassau et al. [6] observed at 4 MHz the formation of hexagonal shapes in a heptagonal cell using microspheres, emulsions and lipid-coated microbubbles (Sonovue${}^{\mathrm{TM}}$: mean diameter 3 μm, with 95% of the bubbles smaller than 10 μm). Phospholipid-shelled microbubbles (diameters 1–10 μm, measured resonance: 3.8 MHz) were also studied by Raiton et al. [9], who reported their accumulation in a low acoustic pressure region at 7 MHz. In all these studies, the geometry of the acoustic pressure and its absolute value were not simultaneously controlled, and this did not allow rheological studies on the shell or the quantitative analysis of bubble-bubble interactions reported here.

Here, we base our analysis on the analytical expressions for secondary Bjerknes forces proposed by Silva and Bruus [17], who modelled the interaction between small spherical particles suspended in an ideal fluid and an external acoustic wave, building on previous results [21,46]. In particular, in Table 1, we report the results of their analysis (up to the first scattering order) under conditions relevant for this study: bubbles or particles aggregating in a standing wave, when the inter-particle distance ϱ is much smaller than the wavelength (kϱ≪1, i.e., in the Rayleigh limit) and the driving frequency is much smaller than the resonance frequency of the bubbles. In Table 1, $\varrho_{∥}$ is the inter-particle distance in the direction of motion, $\varrho_{⊥}$ is the inter-particle distance in the transverse plane, *k* is the wave number and $f_{0}=1-\tilde{\kappa }$ and $f_{1}=2\left(\tilde{\rho }-1\right)/\left(2\tilde{\rho }+1\right)$ are the compressibility and density factors.

According to the analysis in Table 1:

- for bubbles, the forces are attractive in both directions and $\propto \varrho^{-2}$, with the same multiplying coefficient for interactions in the direction of motion and perpendicular to it;
- for particles, forces are repulsive in the direction of motion and attractive in the transverse plane, $\propto \varrho^{-4}$ in both directions, but with a different multiplying coefficient;
- the forces on particles depend on the density ratios $\tilde{\rho }$, while the forces on microbubbles depend on the ratio of compressibilities $\tilde{\kappa }$ and on the driving frequency.

Garbin et al. [47] used optical tweezers to position bubbles of different diameters at a known separation before exciting them with an acoustic pulse, using a high speed camera to monitor their translation. These authors show that the role of the unsteady component of the drag (the “history force”) is crucial for a correct description of the instantaneous translation of coated microbubbles subject to acoustic pulses, but mainly at the start of the motion. As our measurements will be done when the motion of the bubbles is already established, we will neglect this correction.

### 2.4. The Role of Shell Parameters

In the case of phospholipid-coated bubbles, Emmer et al. [25] showed that the linear oscillator model behind Equation (1) is only valid above a threshold pressure, which they related to the onset of volume oscillations, highlighting how smaller bubbles have a larger threshold. According to Doinikov and Bouakaz [30], this behaviour would be due to the shear stress $\tau_{0}$ of the phospholipid as a material: before oscillations start, the acoustic pressure would need to overcome the value:

(3) $$S_{0}=\tau_{0}\left(d_{s}/R_{0}\right)$$

where $R_{0}$ is the equilibrium radius of the bubble and $d_{s}$ is the average thickness of the shell. At a fixed driving acoustic pressure, then, only the largest microbubbles would oscillate: the number of oscillating bubbles would increase with pressure.

The need to explain experimental results (e.g., the asymmetric response of the diameter to repeated cycles of compression/expansion) introduced for phospholipid-coated MBs more complex models [24,26], similar to the ones used for spherical shells in continuum mechanics, adding to the picture two threshold pressures: one for buckling and one for break-up. According to these descriptions, polymer-coated bubbles would maintain a spherical shell at low deflations (elastic state, with the elasticity parameter χ given by the flat sheet formula), but would buckle into spherical caps as the pressure overcomes a threshold given by [48]:

(4) $$p_{\mathrm{buckling}}=C_{\mathrm{buck}}\cdot E_{s}{\left(\frac{d_{s}}{R_{0}}\right)}^{2}$$

where $R_{0}$ is the equilibrium radius of the bubble and $C_{\mathrm{buck}}$ is a coefficient of proportionality. As the driving pressure is further increased, axial symmetry would break through polygonal deformation of the depression, until the pressure is enough to rupture the shell.

For thin-shelled spheres subject to uniform load, the most used coefficient of proportionality in Equation (4) is $C_{\mathrm{buck}}=2/\sqrt{3(1-\nu^{2})}$: an expression attributed to Zoelly [49,50]. It is however known that the presence of axis-symmetric dimple imperfections may reduce the experimental value to approximately 15–20% of the theoretical value for a ”perfect” shell [50,51] i.e., one without imperfections.

The first attempt to estimate shell parameters (and therefore, the threshold pressures determining the different dynamical regimes) is by Gorce et al. [52], who used attenuation and scattering measurements from suspensions of microbubbles. Such measurements, however, may not be valid for predicting the behaviour of each individual microbubble with its specific size and shell thickness. At the single-bubble level, Acoustic Force Microscopy (AFM) has been used to measure the elastic properties of lipid-coated microbubbles with different shell architectures attached to a functionalised substrate [53,54,55], giving Young’s moduli of ∼10 MPa (i.e., much smaller than the values in [34,35]). It has been argued, however, that bubbles attached to a surface may not respond as they would during sonication [56].

Given this uncertainty, during this study, we decided to obtain a direct estimate of shell thickness and buckling pressure, using an Auriga Focussed Ion Beam Scanning Electron Microscope (Zeiss, Cambridge, UK). First, Expancel${}^{\mathrm{TM}}$ bubbles were milled to reveal the average thickness/radius ratio for the sample microbubbles. For this measurement, we deposited a small droplet of the Expancel/SDS solution on an aluminium stub and allowed the water to evaporate. The collection of bubbles left on the stub were then examined using a 2-kV electron beam: an intensity sufficiently low to avoid the need for a conductive coating. A range of ion beam currents was tested to determine the optimal setting at which a section could be cut through the thin shell of the MBs, without causing excessive distortion or obvious damage to the shell itself. A 30-kV/50-pA ion beam was found optimum for this procedure.

To estimate the buckling pressure, we then used a 5 μm-diameter flat tip nano-indenter (ASMEC UNAT-SEM2) and observed the compression behaviour of Expancel${}^{\mathrm{TM}}$ bubbles on a silicon substrate. The indenter was operated in situ inside the Carl Zeiss Auriga 60 FIB-SEM (see above); the stage was tilted to $8^{\circ }$ and sample surface imaged at $82^{\circ }$ using the SEM, at an accelerating voltage of 10 kV. The indenter was positioned manually, centrally and approximately 2 μm above each bubble, with a maximum displacement defined depending on the diameter of the bubble. Bubble diameters were measured only in the horizontal axis to avoid inaccuracies due to image drift and image tilt. The displacement rate of the tests was optimised at 250 nm/s, which was slow enough to record the progress of the test with SEM imaging in real time. Load, displacement, time data and a movie were acquired for each bubble.

## 3. Results

As discussed in Section 2, the results presented in this section were obtained under constant temperature ($20\pm 1^{\circ }$C), with the amplifier working in linear conditions and at concentrations Ξ sufficiently low so as not to alter the speed of sound with the presence of bubbles [23]. We also selected experimental conditions where streaming-induced velocities could be neglected. In summary, the driving acoustic field in the microfluidic device, $p_{a}$, was negligibly perturbed by the bubbles.

### 3.1. Isolated Bubbles

**Figure 2:**
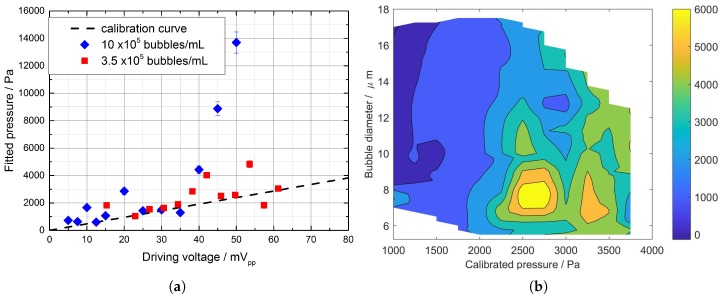
Effective pressure measured from bubble tracks ($p_{\mathrm{meas}}$) as a function of (**a**) driving voltage and concentration, for Expancel${}^{\mathrm{TM}}$ bubbles with a diameter of 10±2 μm (calibration curve from [27]). Also reported in (**b**) is the dependence of $p_{\mathrm{meas}}$ on calibrated pressure ($p_{a}$), over a larger range of bubble diameters, at $3\times 10^{5}$ bubbles/mL.

Figure 2a reports the value of $p_{\mathrm{meas}}$ (averaged over at least five bubble trajectories) as a function of the driving voltage for two different concentrations: $3.5\pm 0.7\times 10^{5}$ bubbles/mL (squares) and $10\pm 2\times 10^{5}$ bubbles/mL (diamonds). The present data, relative to selected Expancel${}^{\mathrm{TM}}$ bubbles of a diameter of 11±1 μm, are compared in this graph with the calibration curve of $47.8\pm 0.8\;\mathrm{Pa}\cdot {\mathrm{mV}}_{\mathrm{pp}}^{-1}$, obtained with solid particles/laser vibrometry/optical tweezers by Memoli et al. [27]. In agreement with the results in [27], for pressures $p_{a}$ below 1.5 kPa (i.e., input voltages below 30 mV), the pressure $p_{\mathrm{meas}}$ determined in this study followed the calibration curve at both concentrations. As the input voltage increased further, however, so did the difference between $p_{\mathrm{meas}}$ and $p_{a}$: the tracked bubbles experienced a stronger force than the equivalent iso-butane spherical particles (i.e. the "uncoated" bubbles), already at $3.5\pm 0.7\times 10^{5}$ bubbles/mL.

We registered a more pronounced difference from the linear calibration when we increased the Expancel${}^{\mathrm{TM}}$ number concentration to $10\pm 2\times 10^{5}$ bubbles/mL: for driving voltages greater than 35 mV${}_{\mathrm{pp}}$, the fitting procedure repeatedly resulted in effective pressures much higher than the ones obtained during the experiments with the lower number concentration (see Figure 2a). In addition, at $10^{6}$ bubbles/mL, the trend of the pressure $p_{\mathrm{meas}}$ determined from bubble trajectories repeatedly appeared more complex than at $3.5\times 10^{5}$ bubbles/mL: as the driving voltage was increased, we observed a local maximum (corresponding to 950 Pa at 20 mV${}_{\mathrm{pp}}$), a minimum and a second (steeper) increase for pressures above 1700 Pa. In brief, Figure 2a presents some evidence of a threshold pressure: we observed deviations from the calibrated input pressure at voltages above 35 mV${}_{\mathrm{pp}}$ (i.e., with the threshold between 1.7 ± 0.5 kPa and 2.0 ± 0.3 kPa).

In order to isolate contributions to this effect due to the different bubble diameters, we ran a second set of measurements at the lowest concentration (i.e., $3\times 10^{5}$ bubbles/mL), but over a larger diameter range. Figure 2b reports $p_{\mathrm{meas}}$ in terms of the pressure $p_{a}$ and the bubble diameter: for a given input pressure $p_{a}$, the pressure measured from trajectories ($p_{\mathrm{meas}}$) decreased with increasing diameter. We explain this effect by noting that, according to Equation (1), the resonance frequency of the tracked bubble $f_{s}$ becomes closer to the driving frequency of 164.33 kHz as the diameter increases (see Appendix A). Using the shell parameters described above in Equations (1) and (2) and using $\beta_{\mathrm{tot}}=0.16\cdots 1.6$ MHz [20,39], a 40% change in the diameter (e.g., from 11 μm–16 μm, as in Figure 2b) is expected to produce a change in the acoustic force potentially as large as 70% (a ratio similar to the one observed in Figure 2b). This effect was mitigated by selecting the bubbles used for tracking, so that for each input pressure $p_{a}$, their diameter was within 2 μm of the mode diameter 10.1 μm and, averaging over the selected bubbles, the resulting values of the fitting parameter. This effect will be neglected in the rest of this work and left to future studies, where we will consider pre-filtering the microbubbles for isolating a more monodisperse population (e.g., using the acoustofluidics methods by Devendran et al. [57]).

### 3.2. Bubble Dynamics towards Aggregation: Doublets

**Figure 3:**
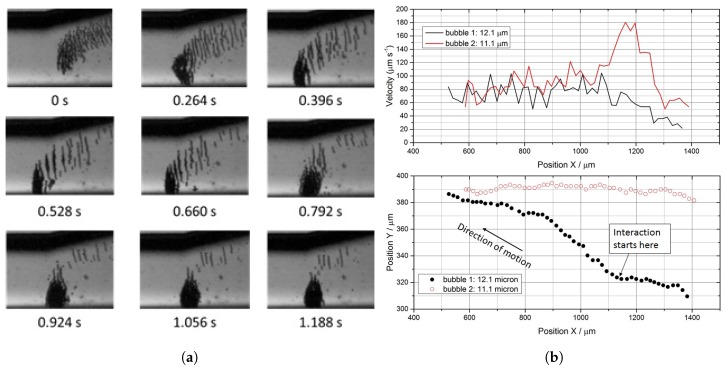
Trap formation at 164.33 kHz, highlighting (**a**) the presence of sub-wavelength structures during aggregation at $p_{a}=$ 1.5 kPa and (**b**) the change in velocity and trajectory due to secondary Bjerknes forces for two bubbles of similar diameter, during doublet formation at $p_{a}=$ 2.7 kPa. In order to clarify that the motion happens in a standing wave, it should be noted that the transducer is positioned far away, towards the top-left of (a).

A more thorough analysis of trap formation showed that, as voltage increased, the process of aggregation could be divided into different steps. First Expancel${}^{\mathrm{TM}}$ isolated microbubbles interacted in pairs, with two bubbles joining into a single entity (i.e., a “doublet”) before proceeding towards the aggregation site, then doublets aggregated into linear structures perpendicular to the direction of propagation of the acoustic field (Figure 3a). These linear structures travelled across the microfluidic chip towards an aggregation point, merging into a larger cloud.

In this section, we repeat the voltage experiment at 164.33 kHz, using MTrackJ to look at bubble dynamics as a function of applied voltage. This time, however, we look for pairs of similarly-sized Expancel${}^{\mathrm{TM}}$ microbubbles combining to form a non-coalescing doublet and measure their diameter and velocity along the path. As described by Garcia-Sabatè et al. [18], who calculated the secondary Bjerknes forces in an acoustic levitator using monodisperse latex beads, two isolated particles starting to interact (at time $t_{d_{c}}$) see a net change in their velocity, preceded and followed by a period of almost constant velocity (see Figure 3b). In these periods, when acceleration can be neglected, the drag force can be considered equal to the total force acting on each bubble, and the interaction force can therefore be calculated from the difference $F_{\mathrm{interaction}}=F_{\mathrm{after}}-F_{\mathrm{before}}$ and interpreted using the inter-bubble distance $d_{c}$, measured at $t_{d_{c}}$ (see also Appendix B). Expancel’s wide size distribution made it extremely difficult to find pairs of similarly-sized microbubbles within our setup: we managed to identify only a very limited number of such pairing events during post-processing (i.e., a maximum of three for each 30-s movie, relative to a set of experimental parameters), and at 164.33 kHz, we observed doublet formation only for input voltages above 25 mV${}_{\mathrm{pp}}$ (i.e., ∼ 1.2 kPa). Garcia-Sabatè et al. [18], who reported 17 useful pairs over two hours of recordings, suffered a similar scarcity of analysable data.

**Figure 4:**
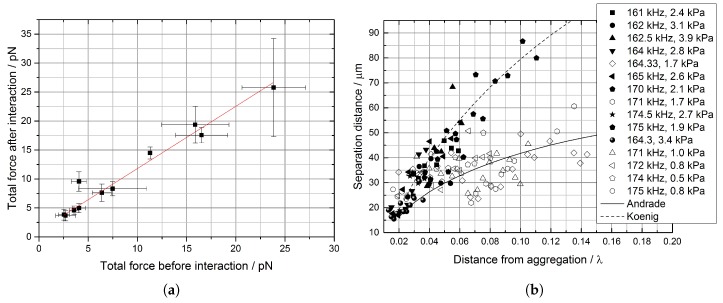
Measurements during aggregation: (**a**) Stokes’ drag $F_{D}=6\pi a\mu v$, calculated before and after interaction; (**b**) Spacing at different frequencies and pressures. The spacing in (b) was measured as a function of the distance from the aggregation point (in units of the wavelength) at different frequencies and pressures. Also reported are two fitting lines, obtained using Equation (5), with the coefficients from [28,29]. Details of the fitting constants are in the text.

Figure 4a reports the drag calculated using Stokes’ formula $F_{\mathrm{drag}}$ before and after interaction for pairs of bubbles forming a doublet at different input voltages and frequencies. Data in Figure 4a are relative to bubbles with diameters between 10±0.2 μm and 14.3±0.2 μm and an average number concentration of $3.5\times 10^{5}$ bubbles/mL. An increase of the total force acting on the bubbles, after interaction started, was observed at all values of the voltage and for each diameter, with the difference between the two values attributed to secondary Bjerknes forces acting perpendicularly to the direction of motion. The linear fit in Figure 4a (least squares with $R^{2}=0.94$) has a slope of 1.07±0.08, meaning that the radiation force acting on the single bubbles only changes during the interaction by a term $F_{\mathrm{interaction}}$ of the order of 1.0±0.6 pN (i.e., the intercept in Figure 4a).

Since 1 pN is at the limit of our resolution (hence the large uncertainty), a larger number of suitable events would be needed to conclude without a doubt whether our findings agree with the dependence of $F_{\mathrm{interaction}}$ on inter-bubble distance $\varrho_{⊥}$ and on bubble diameter *a* from Table 1. Our preliminary analysis, however, confirms the sign of the force ($F_{\mathrm{after}}>F_{\mathrm{before}}$) and the dependence $∼a^{6}\;\varrho_{⊥}^{-2}$ expected from bubbles (see Appendix B), thus providing an experimental proof to the modelling of [17], at least in the direction perpendicular to the motion. If confirmed by further measurements, the result in this section will strengthen the conclusions that bubbles cannot be treated as particles, for which the dependence would instead be $∼a^{6}\;\varrho_{⊥}^{-4}$.

### 3.3. Ripple Formation

For the data presented here, we injected Expancel${}^{\mathrm{TM}}$ bubbles prepared as described in Section 2 and drove the transducer at different frequencies, but with a common input voltage of 40 mV${}_{\mathrm{pp}}$. In our experiments, we observed the formation of sub-wavelength structures at different frequencies (see Appendix C). Figure 4b reports on the vertical axis the separation between the *r*-th and (r+1)-th “ripple” ($\xi_{r,r+1}$), determined using ImageJ along the imaginary line connecting the centre of the most distant ripple (typically the most regular one) and the centre of the aggregation. The horizontal axis reports the distance of the ripple from the antinode (*y*) in units of the wavelength, to facilitate comparisons between different frequencies. Uncertainties in the line separation were always larger than 2 μm (i.e., 10-times the pixel resolution of 0.2 μm) and below 3 μm, so we conservatively assumed this value for all the points. The uncertainty on the distance from the node was estimated at 5%.

In order to interpret our results, we assumed for each frequency a sinusoidal field along the imaginary line above, where we observed ripple formation. Under this hypothesis, the first node was at $y_{0}=\lambda /4$ (i.e., at least 2 mm away, so that all the motion happened within the capture area of the aggregation point). Following the example of Robinson [45], but using the attractive force for bubbles from Table 1, we looked for a correlation linking the spacing between ripples with the distance from the closest aggregation point for bubbles at the selected frequency, but we found the same expression obtained in the literature for particles [28,29,45]:

(5) $$\xi_{r,r+1}=C_{0}{\left(\cos\left(\pi \frac{y}{y_{0}}\right)\right)}^{s}$$

where $C_{0}$ is the distance between the first two ripples, while s=2/3 is the exponent proposed by Koenig [28,45] (for which interactions are due to elastic response) and s=0.44 according [29] (for which ripples are created by local microstreaming). According to Equation (5), the spacing does not depend on the acoustic pressure, but only on y/λ, where *y* is the distance from the aggregation point. We concluded that ripple formation cannot be used to distinguish the sign of the force along $\varrho_{∥}$ (and thus to distinguish bubbles and particles, according to Table 1).

Our findings, however, highlight a pressure-dependent behaviour for the spacing between ripples. Figure 4b presents two fitting lines, one using s=2/3 according to Koenig [28,45] and one with s=0.44 according to da C. Andrade [29]. As shown in Figure 4b, close to the aggregation point, it is not possible to distinguish whether data follow one correlation or the other; however, as ripples are considered further away, a significant difference can be observed, and two trends can be highlighted. Fitting data with Equation (5) showed that bubbles moving under higher driving pressures followed Koenig’s correlation, while the others followed the relation in [29]. The fitting coefficients in Figure 4b were obtained using a least-squares method: $C_{\mathrm{Koenig}}=120\pm 10$ μm and $C_{\mathrm{Andrade}}=55\pm 8$ μm, respectively. For input pressures above 1.9 kPa, then, the initial spacing was almost twice as large as in the case of lower pressures: again, a threshold behaviour (different frequencies give different pressures for the same voltage in our setup; see also Section 2). Given that [45] and [29] gave a different explanation for their observations, a change in the correlation followed may be indicative of a change in the dynamics. This will be discussed further in Section 4.

### 3.4. Direct Estimation of Shell Parameters

**Figure 5:**
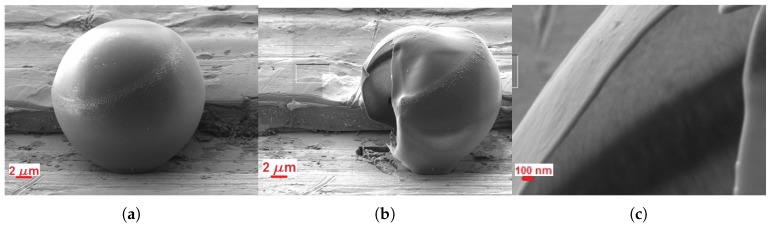
A 22-μm Expancel${}^{\mathrm{TM}}$ bubble during milling with the FIB-SEM. (**a**) Before; (**b**) After; (**c**) Zoom after milling.

Figure 5a shows a 22-μm Expancel${}^{\mathrm{TM}}$ bubble, before milling. The ring, offset from the equatorial plane, is a feature that we observed on all the sampled bubbles: it appeared to divide a smaller, smoother region from a larger, rougher surface. Figure 5b shows the sphere after sectioning, with Figure 5c, a higher magnification image of the shell cross-section produced from the top part of Figure 5b. The upper, smoother part of the sphere appeared to have distorted more, after relieving the internal pressure in the sphere by cutting the shell. The action of milling also appeared to cause some flaring outward of the shell edge, making accurate measurement of the cross-section difficult in the upper part of the shell. Measurements of the shell section were then taken in the lower part of the shell, giving a ratio $d_{s}/R_{0}=6.3\pm 0.9\times 10^{-3}$ for a 22-μm bubble. A similar section through a bubble of 27 μm in diameter gave a ratio of $3.3\pm 0.3\times 10^{-3}$.

**Figure 6:**
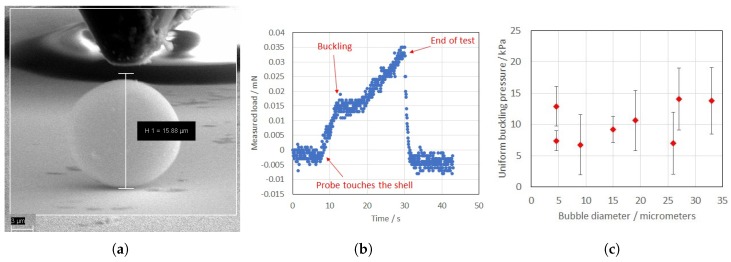
Measurements of the uniform buckling pressure under a scanning electron microscope: (**a**) A view of the measurement setup for a 15-μm Expancel${}^{\mathrm{TM}}$ bubble as a result of the buckling pressure; (**b**) Measured load on the same bubble as a function of time, at a loading rate of 250 nm/s; and (**c**) Buckling pressures for different bubble diameters, reported for the uniform condition.

Figure 6a shows the probe at the start of the measurement, before it was driven towards the bubble at a constant speed (see Supplementary Movie Video S3). Measuring the load on the nano-indenter gave results like the those in Figure 6b: for a given bubble, the load increased linearly with the displacement until buckling occurred, then the load remained constant until the sides of the tip hit the bubble again. Afterwards, the load started increasing again (with a lower rate), until the measurement was terminated (see the additional movie). Weighting the measured “buckling load” with the surface of the tip gave the pressure locally applied on the bubble. Figure 6c reports the experimental pressures, which have been weighted with the geometrical factor $G=6/[\pi \alpha \left(6-\alpha^{2}\right)]$, as suggested by [58], where α is the angle between the radius passing through the end of the tip and the radius passing through the centre of the tip (i.e., the angle subtended by the inversed region to the centre). According to [58], this correction can be used to compare the experimental situation of concentrated load (i.e., like the one we applied) with the theoretical one of uniform load. With this correction, the values in Figure 6c give a weighted average of $p_{\mathrm{buckling}}^{exp}=9\pm 1$ kPa as the value for a perfect sphere.

Assuming the properties above—i.e., $C_{\mathrm{buck}}=0.605$ and an average value of $d_{s}/R_{0}=5.0\pm 0.9\times 10^{-3}$ for all bubble diameters in Equation (4)—this measurement of $p_{\mathrm{buckling}}^{\exp}$ is compatible with $E_{s}^{\exp}=3\times 10^{8}$ Pa (i.e., 1/10th of the literature value from [34,35], 10-times larger than the values by AFM [53,54,55]).

The presence of a welded joint in Figure 5a, however, may lead to much lower experimental values under uniform load conditions (i.e., like the ones in an acoustofluidic device). In the presence of similar imperfections, Zhang et al. [51] have measured on metallic shells values as low as 15% of the theoretical value given by Equation (4). Similar effects have been reported by others [50,56]. In this context, the threshold we observed between 1.7 ± 0.5 kPa and 2.0 ± 0.3 kPa (@164.33 kHz) is consistent with the onset of buckling. Future studies (benchmarked by high speed imaging) will be instrumental to confirm the nature of the observed threshold.

## 4. Discussion

In this work, we monitored the dynamics of Expancel${}^{\mathrm{TM}}$ bubbles, as they aggregated in the well-characterised acoustic field of an acoustofluidic chip. First, we observed a difference between the pressures obtained by bubble tracking ($p_{\mathrm{meas}}$) and the input pressures ($p_{a}$) measured by other methods, which becomes evident for input voltages above 35 mV${}_{\mathrm{pp}}$ (corresponding to pressures above 1.7 kPa and concentrations of the order of $10^{6}$ bubbles/mL). Above this threshold, we repeatedly observed the formation of doublets, the appearance of ripples during trap formation and a pronounced dependence on bubble number concentration: all phenomena previously observed with particles and classically attributed to the presence of secondary Bjerknes forces, due to volume oscillations. We also observed a dependence of the force per unit volume on bubble diameter and, above the threshold, a change in the law that describes the mutual distance between ripples: effects not clearly explained by the description of secondary Bjerknes forces for solid particles. Having excluded the role of non-linearity in the E&Iamplifier or minor changes of temperature in the glass chip (see Section 2), we investigate in this section other potential causes to explain our observations.

Since the data in Figure 2a are averaged over bubble diameters far from their resonance, the weighting factor in Equation (2) can be considered constant, leaving an effect that depends on the number concentration and therefore on the average bubble-bubble distance. At low concentrations, we explain this effect with the presence of secondary Bjerknes forces. With a derivation similar to the one in Robinson [45], we consider three bubbles/particles moving towards the aggregation point, positioned on a line at distances $x-\varrho_{∥}$, *x* and $x+\varrho_{∥}$ from the aggregation. We calculate the force on the central one by inserting the expressions for $F_{∥}$ and $F_{⊥}$ from Table 1 in the force balance utilised to analyse particles/bubble tracks and obtain:

(6) $$\begin{array}{ccc} \begin{array}{c} \mathrm{particles}\\ \left(f_{1}\gg f_{0}\right) \end{array} & = & \left\{ \begin{array}{ccc} \dot{s} & = & Ap_{a}^{2}\left(\Phi k-f_{1}^{2}\frac{a^{3}}{\varrho_{∥}^{4}}\sin\left(2k\varrho_{∥}\right)\right)\times \\ & & \sin\left(2ks\right)\\ \dot{t} & = & -\frac{9}{4}Ap_{a}^{2}\left(f_{1}^{2}\frac{a^{3}}{\varrho_{⊥}^{4}}\right)\sin\left(2ks\right) \end{array}\right. \end{array}$$

(7) $$\begin{array}{ccc} \begin{array}{c} \mathrm{bubbles}\\ \left(f_{1}\ll f_{0}\right) \end{array} & = & \left\{ \begin{array}{ccc} \dot{s} & = & Ap_{a}^{2}\left(\Phi k+\frac{a^{3}f_{o}^{2}}{3\varrho_{∥}^{2}}k^{2}\sin\left(2k\varrho_{∥}\right)\right)\times \\ & & \sin\left(2ks\right)\\ \dot{t} & = & -Ap_{a}^{2}\left(\frac{a^{3}f_{o}^{2}}{3\varrho_{⊥}^{2}}k^{2}\right)\sin\left(2ks\right) \end{array}\right. \end{array}$$

(8) $$\begin{array}{ccc} A & = & \frac{1}{9\eta_{l}}\frac{a^{2}}{\rho_{l}c_{l}^{2}} \end{array}$$

where *s* is the coordinate along the direction of motion (s=0 at the aggregation point) and *t* is the coordinate perpendicular to it. The second term on the RHS of Equations (6) and (7) represents the contribution to the velocity, respectively for particles and bubbles, due to the bubble/particles preceding and following the selected one, both at distance $\varrho_{∥}$ along the direction of motion.

Equations (6) and (7) show that, when s≪λ and $\varrho_{∥}\ll \lambda$, the presence of secondary Bjerknes forces can be described by adding a term $\tilde{\Phi }$ to the standard acoustic contrast factor: for the same fixed input pressure $p_{a}$, interacting bubbles would experience forces greater than the ones acting on solid particles with the same $\varrho_{∥}$ and number concentration. Assuming as average inter-bubble distance the Wigner–Seitz radius $\varrho_{∥}={\left(3/\left(4\pi n\right)\right)}^{1/3}$ where *n* is the number concentration, the expected additive correction to the acoustic contrast factor of a 10-μm diameter bubble at 164.33 kHz is $\tilde{\Phi }/\left(\Phi k\right)=a^{3}f_{0}^{2}k^{2}/\left(3\varrho_{∥}^{2}\right)\approx 0.20$ for $n=3.5\times 10^{5}$ bubbles/mL and $\tilde{\Phi }/\left(\Phi k\right)\approx 0.44$ for $n=10\times 10^{5}$ bubbles/mL.

We confirmed the validity of this result by fitting a line to the averaged data at $3.5\times 10^{5}$ bubbles/mL in Figure 2a: a least-squares fit gives a coefficient of $58.5\pm 0.8\;\mathrm{Pa}\cdot {\mathrm{mV}}_{\mathrm{pp}}$ (1 standard deviation) with an $R^{2}=0.8$ and a value of $\tilde{\Phi }\approx 0.22\pm 0.04$. When passing to $10^{6}$ bubbles/mL, however, the trend in Figure 2a is far from linear: other factors need to be taken into account.

**Figure 7:**
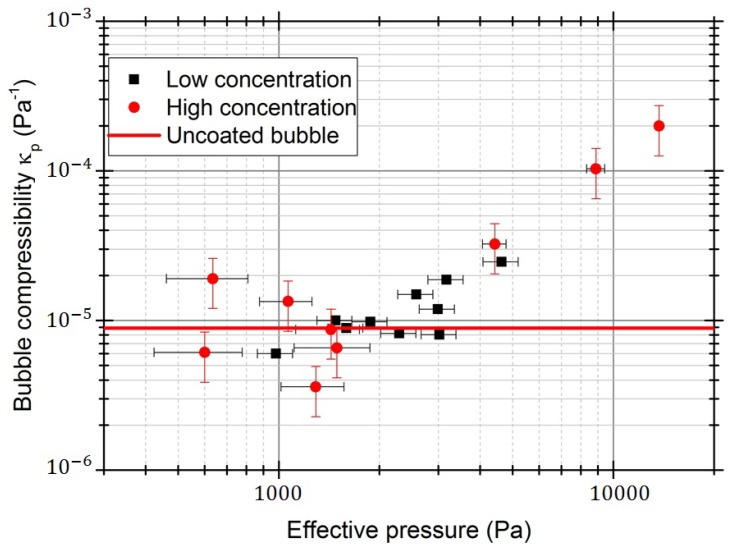
Expancel${}^{\mathrm{TM}}$ compressibility measured in this study, at different bubble concentrations, reported as a function of the effective pressure $p_{\mathrm{meas}}$. Also reported is the value of $\kappa_{p}$ for an uncoated iso-butane bubble.

In order to estimate the effect of the shell, we use the observed trajectories to measure microbubble compressibility, as in [3], after correcting Φ for the effect of interactions $\tilde{\Phi }$. In Figure 7, we report the values of $\kappa_{p}=-V^{-1}\left(\partial V/\partial p\right)$ as a function of the measured pressure from the data in Figure 2a. Also reported is the compressibility of an uncoated bubble (i.e., only made of iso-butane, with Φ=−6753 at 20 ${}^{\circ }$C). In this way, we account for the dependence on concentration by noting that secondary Bjerknes forces modify the acoustic pressure locally acting on the bubbles: compressibility should be related not to $p_{a}$, but to the effective pressure $p_{\mathrm{meas}}$ from Figure 2a. Figure 7, which presents compressibility $\kappa_{p}$ as a function of effective pressure, allows the threshold to be estimated between 1.7 ± 0.5 kPa and 2.0 ± 0.3 kPa (164.33 kHz).

The observed (quadratic) trend with increasing pressure is not compatible with the onset of oscillations, as described by Emmer et al. [25] and Doinikov and Bouakaz [30], but instead agrees with the models of shell compressibility based on Hooke’s law [41,49], which account for buckling. Paul et al. [59], in particular, proposes near the transition to buckling a quadratic dependence of the radial compression rate $R/R_{0}$, where $R_{0}$ is the equilibrium radius of the bubble. For pressures above the threshold for the onset of oscillations, where the radial compression rate increases linearly with pressure [25], this would correspond to a quadratic dependence on pressure.

The different behaviour observed in Figure 4b, for a similar threshold pressure of 1.7 kPa, confirms the interpretation of this dynamical change as the onset of buckling. We already highlighted that all the data following da C. Andrade’s correlation are relative to acoustic pressures below 1.7±0.3 kPa, while the ones following Robinson’s relation are relative to pressures above 1.9±0.3 kPa. Looking back at the original studies by these authors, we notice that they propose two different mechanisms of interaction. According to da C. Andrade, the formation of lines perpendicular to the direction of motion in an air-filled Kundt tube is due to the “circulation of air between two particles, due to the vibration of the involved dust particles” [29]. da C. Andrade highlighted this circulation with careful imaging and noted that while “at small distances a vortex system is formed around two particles, pushing them together, at larger distances the two particles tend to repel each other, so that each can form its own vortex system”. In modern terms, da C. Andrade is attributing separation to the presence of acoustically-driven fluid movement between the particles (e.g., like the one caused by repeated buckling). Koenig’s description [45], instead, aligns with the description of secondary Bjerknes forces: interactions between elastic particles that are either attractive or repulsive in nature. This difference in description, if extendible to coated microbubbles, would indicate a different interaction method as the bubbles move through the threshold. Further studies will look at different types and sizes of bubbles, in order to establish a clearer explanation.

It is however expected that this effect will be stronger for lipid-coated microbubbles, which start buckling at very low pressures [26]: with a threshold in the kPa region, MBs subject to the typical pressures used in diagnostics would always buckle. This is a crucial observation, as buckling causes an increase in the sub-harmonic emission during sonication [24,26] that, properly calibrated (e.g., having a precise value for $p_{\mathrm{buckling}}$), could be used for localised blood pressure measurements [60]. In this sense, experiments with phospholipid-coated MBs [61] have found a sub-harmonic increase of 17 dB, when using an external (static) pressure of 60 mmHg (i.e., $p_{\mathrm{buckling}}\leq 8$ kPa).

We also observed that microbubbles tend to aggregate into sub-wavelength structures as their number concentration increases (above $10^{6}$ bubbles/mL, in this study). This may also happen in medical applications, where concentrations of the order of $10^{8}÷10^{9}$ bubbles/mL are typically used [23], but the presence of such phenomena is typically neglected.

With the incremental number of attempts of using MBs for therapeutic applications, experimentation on MBs has moved to novel manufacturing methods (e.g., [62,63,64]) and/or formulations [65,66]. In this context, knowing microbubbles’ behaviour near $p_{\mathrm{buckling}}$ and their compressibility as pressure increases (i.e., the coefficient for the quadratic dependence afterwards) may help practitioners to distinguish how a specific MB responds to sonication, even at higher pressures. Not all the bubble formulations may be equally effective under each delivery method. While some delivery mechanisms rely on the bubble carrier being destroyed, others depend in fact on the drug being propelled through the blood vessel walls [8]: a mechanism still poorly understood, dominated by non-linear oscillations [67], where buckling may have a significant impact. It is however in techniques like sonoporation [68] or sonothrombolysis [69] that differences between buckled/unbuckled bubbles may be determinant. Not only because these techniques are often used at frequencies far from the bubble resonance and relatively lower acoustic pressures (i.e., closer to the experimental conditions used here), but because in these methods the microstreaming induced by buckling or the presence of microstructures may have a directional effect not accounted for so far [70].

The microchip used in this study offers a privileged environment to optimise delivery parameters and/or to understand better the biophysical phenomena underpinning microbubble-mediated drug delivery with different potential vectors, but like other “in vitro” set-ups [71], it is still far from the environmental conditions microbubbles experience in a patient. Future studies will consider simple upgrades, like adding a flow rate and/or changing the operating temperatures, and more complex ones, like functionalising the glass walls with more tissue-relevant substrata. In parallel, we will look at ways to incorporate characterisation setups (like ours) into existing microfluidic-based systems for microbubble production, for in situ quality assurance.

## 5. Conclusions

In this work, we have measured the primary and secondary Bjerknes forces acting on polymer-coated microbubbles in an acoustofluidic chip, excited below (“to the red of”) their resonance. Due to the presence of the shell, we observed that coated microbubbles cannot be simply treated as particles in such an environment, but that they exhibit a clear threshold in their compressibility as pressure is increased. Thanks to experiments at different number concentrations of the bubbles in the chip, benchmarked by measurements under an electron scanning microscope, we provide evidence for this threshold to be identified as the onset of buckling. We conclude that the presence of the shell does not influence Bjerknes forces before buckling starts, i.e., below the buckling threshold, bubbles in aqueous fluids can be treated as particles with a negative acoustic contrast factor, neglecting their shell. This simple result may be useful to other researchers, conducting acoustofluidic experiments with bubbles, elastic particles or shelled vesicles (e.g., liposomes).

In acoustofluidic applications, where oscillating bubbles are used for acoustically-driven microflow control [11,13], our results show that local microstreaming may be enhanced by the presence of buckling, after the threshold (Section 3.3). Off-resonance, actuation may even be triggered by modifying the amplitude of the driving acoustic pressure, instead its frequency, with cycles of hysteresis across the buckling threshold. Long-lasting coated bubbles, like the ones suggested by Bertin et al. [13], may therefore be better placed to exploit these effects.

In the medical context, where lipid-coated microbubbles are used for diagnostic and (incrementally) for therapeutic applications, our methods may lead to precise measurements of the compressibility of different bubble vectors, each manufactured with a different method, helping practitioners in choosing the “right bubble” for a specific drug delivery task. In the long term, measurements like the ones presented in this study may reduce the duration of clinical studies, by providing a standardised test for comparing novel microbubble formulations with already approved ones.

In this study, we used a direct estimate of the shell thickness and of the onset of buckling to determine the elastic properties of the material in the shell. If the latter are known, as is the case for the lipids typically going on the shell of medical microbubbles, precise knowledge of the buckling pressure may be linked to the average shell thickness, and therefore to the average payload of a bubble: a key measurement underpinning “dose” planning during microbubble-based treatments and the taste experience for bubbles in food [72].

## Figures and Tables

**Table 1:** Forces acting on bubbles/particles in a standing wave according to [17,21,46], with kϱ≪1, and $E_{0}=p_{a}^{2}/\left(2\rho_{l}c_{l}^{2}\right)$ is the acoustic energy density. A positive sign indicates a repulsive force, while a negative sign indicates attraction. Here, *k* is the wave number, *a* is the radius of the particle/bubble, $\varrho_{∥}$ is the inter-particle distance in the direction of motion, $\varrho_{⊥}$ is the inter-particle distance in the transverse plane, *k* is the wave number, $f_{0}=1-\tilde{\kappa }$ and $f_{1}=2\left(\tilde{\rho }-1\right)/\left(2\tilde{\rho }+1\right)$ are the compressibility and density factors.

Object	Force in the Direction of Motion $F_{∥}$	Force in the Transverse Plane ($F_{⊥}$), when kh≪1
particles ($f_{1}\gg f_{0}$)	$+\frac{4\pi }{3}E_{0}f_{1}^{2}a^{6}\varrho_{∥}^{-4}\cos^{2}\left(kh\right)$	$-3\pi E_{0}f_{1}^{2}a^{6}\varrho_{⊥}^{-4}\sin\left(2k\varrho_{∥}\right)$
bubbles ($f_{1}\ll f_{0}$)	$-\frac{4\pi }{9}E_{0}f_{0}^{2}k^{2}a^{6}\varrho_{∥}^{-2}\cos^{2}\left(kh\right)$	$-\frac{4\pi }{9}E_{0}f_{0}^{2}k^{2}a^{6}\varrho_{∥}^{-2}\sin\left(2k\varrho_{⊥}\right)$

**Figure A1:**
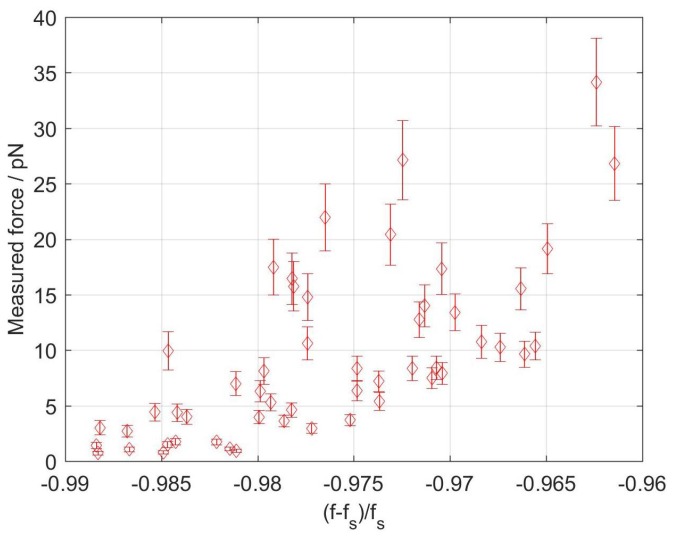
The measured pressures $p_{\mathrm{meas}}$ from Figure 2b, reported as a function of the relative distance between the driving frequency and the resonance frequency calculated from Equation (1).

**Figure A2:**
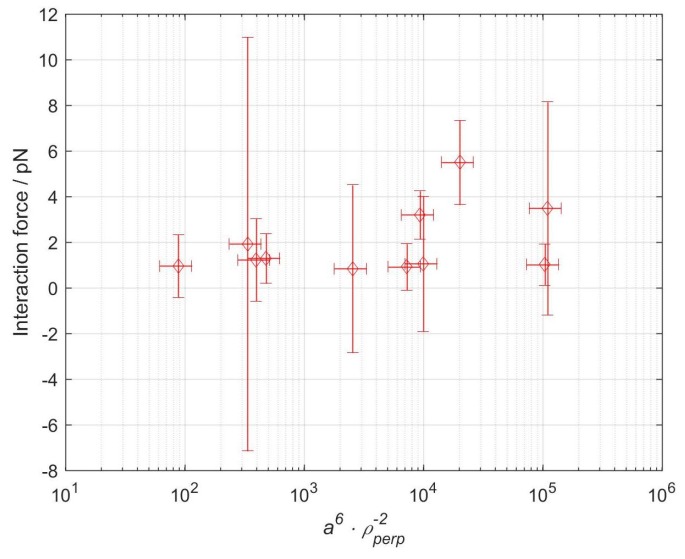
The value of $F_{interaction}=F_{after}-F_{before}$, calculated from Figure 4a, according to the method proposed by Garcia-Sabatè et al. [18].

**Figure A3:**
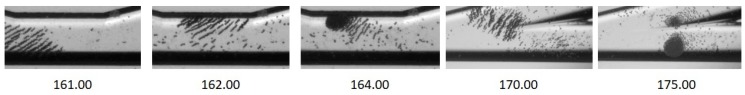
Formation of subwavelength structures during movement of the Expancel${}^{\mathrm{TM}}$ microbubbles in the acoustofluidic chip, in the proximity of an aggregation point. Also reported are the frequencies (in kHz) relative to each image.

## Supplementary Materials

The following are available online at http://www.mdpi.com/2072-666X/9/8/404/s1. Video S1: aggregation at 164.33 kHz, before the threshold (23 mV${}_{\mathrm{pp}}$, 1100 Pa, low concentration). The horizontal line on the top of the channel is 780 μm long; Video S2: aggregation at 164.33 kHz, after the threshold (55 mV${}_{\mathrm{pp}}$, 2630 Pa, low concentration). The horizontal line on the top of the channel is 780 μm long; Video S3: Compression of a 15 μm Expancel${}^{\mathrm{TM}}$ bubble with the nanoindenter.

## Appendix A. Effect of Bubble Diameter on the Measured Force

Figure A1 reports the values of $p_{\mathrm{meas}}$ from Figure 2b, reported as a function of the relative distance between the driving frequency and the resonance frequency calculated from Equation (1). In this picture, the driving frequency is fixed, but changes in the bubble diameter result in a change in the resonance frequency $\omega_{s}$.

## Appendix B. Interaction Force, Calculated According to Garcia-Sabatè et al. [18]

Figure A2 reports the values of $F_{interaction}=F_{after}-F_{before}$, calculated when $p_{\mathrm{meas}}$ > 1.5 kPa at the point where interaction starts, according to the method reported in [18]. Results are compatible with a constant, as a function of $a^{6}\varrho_{⊥}^{-2}$, highlighting a potential agreement between the measurements and the expression of secondary Bjerknes forces attributed to bubbles (see Table 1). Due to the large uncertainties and the scarcity of data, these measurements cannot however be considered a conclusive proof in this direction.

## Appendix C. Visualisation of the Sub-Wavelength Structures

Figure A3 shows the formation of subwavelength structures at different driving frequencies, but with a constant input voltage. Photos were taken in the proximity of aggregation points located all across the chip: since different peaks in Figure 1b correspond to different spatial distributions of the pressure in the chip, the position of the strongest aggregation point changed with frequency.
